# Effect of *Xpcl1* Activation and *p27^Kip1^* Loss on Gene Expression in Murine Lymphoma

**DOI:** 10.1371/journal.pone.0014758

**Published:** 2011-03-11

**Authors:** Daniel A. Kuppers, Harry C. Hwang, Aimee L. Jackson, Peter S. Linsley, Bruce E. Clurman, Matthew L. Fero

**Affiliations:** 1 Clinical Research Division, Fred Hutchinson Cancer Research Center, Seattle, Washington, United States of America; 2 Rosetta Inpharmatics, Seattle, Washington, United States of America; University of Edinburgh, United Kingdom

## Abstract

Mice lacking the *p27^Kip1^* Cdk inhibitor (*Cdkn1b*) exhibit increased susceptibility to lymphomas from the Maloney murine leukemia virus (M-MuLV), and exhibit a high frequency of viral integrations at *Xpcl1* (*Kis2*), a locus on the X-chromosome. *Xpcl1* encodes miR-106a∼363, a cluster of microRNAs that are expressed in response to adjacent retroviral integrations. We report the first large-scale profile of microRNA expression in MuLV-induced lymphomas, in combination with microarray gene expression analysis. The source material was T-cell lymphomas induced by M-MuLV in *p27^Kip1^* knockout mice and normal thymus. Surprisingly, the overall levels of miRNA expression were equivalent in lymphomas and normal thymus. Nonetheless, the expression of specific microRNAs was altered in tumors. The miR-106a∼363 miRNA were over-expressed in lymphomas, particularly those with viral integrations at the *Xpcl1* locus. In contrast, *p27^Kip1^* deletion itself was associated with a different pattern of microRNA expression. Gene expression was dramatically altered in lymphomas, yet paralleled data from T-cell lymphomas induced by other mechanisms. Genes with altered expression in association with the *p27^Kip1^* null genotype were of similar functional classes to those associated with *Xpcl1* integration, but with the opposite pattern of expression. Thus, the effect of *p27^Kip1^* deletion may be to oppose an anti-oncogenic effect of *Xpcl1* rather than enhancing its oncogenic functions. A subset of miR-106a∼363 target genes was consistently reduced in lymphomas with *Xpcl1* integrations, particularly genes with cell cycle and immune functions. We identify four predicted target genes of miR-106a∼363 miRNA, including *N-Myc (Mycn)*, and the TGF-beta receptor (*Tgfbr2*) using 3'UTR reporter assays. Still, bioinformatic miRNA target predictions were poor predictors of altered gene expression in lymphomas with *Xpcl1* integration. Confirmation of miR-106a∼363 gene targeting relevant to the tumor phenotype requires *in vivo* validation, because only a subset of predicted targets are consistently reduced in tumors that overexpress miR-106a∼363.

## Introduction

Mice lacking one or both copies of the *p27^Kip1^* Cdk inhibitor are susceptible to developing a variety of tumor types when exposed to mutagenic agents. [1], [2] Several oncogenes and tumor suppressor genes have been shown to cooperate with *p27^Kip1^* loss, including PTEN and APC, in the induction of breast, colon and prostate cancers in mouse models. [2]–[4] We previously identified *Xpcl1* as a common viral integration site in T-cell lymphomas induced by Moloney murine leukemia virus (M-MuLV) infection. M-MuLV integrations targeted *Xpcl1* at a higher frequently in tumors induced in *p27^Kip1^* knockout mice. This finding suggested that *p27^Kip1^* deletion cooperates with *Xpcl1* activation in tumorigenesis, thus the region was referred to as the *X-chromosome p27 cooperating locus*. [5] Expression of a non-coding RNA from this locus was increased in tumors with *Xpcl1* integrations, and this was higher than levels expressed in normal thymic tissue. *Xpcl1* is not exclusively targeted by M-MuLV in mice with *p27^Kip1^* deletions; it has also been identified as a viral integration site in p27+/− and wildtype animals. [5], [6] Likewise, integrations of other retroviruses, RadLV and SL3-3, have been independently reported to map to the same X-chromosomal site in murine lymphomas. [7], [8] These integrations were associated with increased expression of a cluster of additional non-coding RNA splice variants, proximal to the viral integrations that were collectively referred to as *Kis2*. Mapping of microRNA sequences to the murine and human genomes identified the locus as the coding sequence for a cluster of five microRNAs, commonly referred to as miR-106a∼363, which span the proximal and distal EST clusters. [9] Thus the normal gene, herein referred to as *Xpcl1*, apparently produces a primary transcript that is cleaved to form component pre-miRNA hairpins, as well as the flanking ESTs. Indeed, the individual microRNA comprising miR-106a∼363 have been shown to be increased in tumors with viral integrations at the locus. [8], [10] Large scale viral mutagenesis screens identified increased cointegration of retroviruses at *Xpcl1* and cyclin D3 loci, which further suggests an interaction between the Rb pathway with *Xpcl1* in tumorigenesis. [11]

MicroRNAs are products of RNA precursors transcribed by RNA polymerase II and cleaved by the nuclear microprocessor complex, which is comprised of the RNAse III, Drosha, and DGCR8/Pasha, yielding 50−80 bp stem-loop pre-miRNA (reviewed by R. Shivdasani). [12] Cytoplasmic Dicer further cleaves the hairpin structures off pre-miRNA producing double stranded RNA that includes the mature 20−22 bp microRNA product. Mature miRNA enter the RNA induced silencing complex (RISC), where recognition or target mRNA sequences may lead to translational silencing or degradation of mRNA targets. [13] The miRBase database currently lists 600 microRNAs in the mouse genome. [14] MicroRNA have been shown to impact a wide variety of physiological activities including developmental timing, cell proliferation, cell death, and hematopoiesis through a combination of direct effects on target gene expression and a cascade of secondary effects. [15] The first oncogenic non-protein encoding RNA discovered was *BIC*, which lies at a common integration site of the avian leukosis virus, and encodes miR-155. [16], [17] MiR-155 is overexpressed in a variety of human B-cell lymphomas, and *BIC* overexpression induced lymphomas in transgenic mice. [18]– Human B-cell lymphomas have also been shown to harbor gene rearrangements involving the miR-17∼92 microRNA cluster, which is a paralog of miR-106a∼363. Overexpression of miR-17∼92 in transgenic mice increased the rate of Myc-induced lymphomas and was associated with reduced apoptosis, whereas cells lacking miR-17∼92 are prone to Myc induced apoptosis. [21], [22]

It has been reported that tumor cells exhibit a global reduction in miRNA expression compared to normal tissues, and that impairment of the miRNA processing machinery is oncogenic. [23], [24] Yet, little is known about the global patterns of miRNA or protein-coding gene expression in lymphomas induced by murine retroviruses. A hypothesis of the current work is that genetic alterations of M-MuLV induced lymphomas would result in characteristic phenotypic patterns of miRNA and protein-coding gene expression. Deletion of *p27^Kip1^* has been shown to be associated with more rapid M-MuLV-induced lymphoma development, with a higher frequency of viral integrations at *Xpcl1*. We therefore hypothesized that the gene expression phenotype associated with *p27^Kip1^* deletion in tumors would reflect an enhancement of the tumor phenotype, or an enhancement of the miRNA or gene expression profile specific to *Xpcl1*. The classes of genes associated with *p27^Kip1^* loss or *Xpcl1* activation may give clues to the altered functions associated with these mutations. Herein, we report the results of both a miRNA and a protein-coding gene expression analysis from M-MuLV induced T-cell lymphomas. We also validate the targeting of four genes by miRNA from the miR-106a∼363 cluster.

## Results

### Global miRNA expression in lymphomas vs. normal thymus

**Figure 1:**
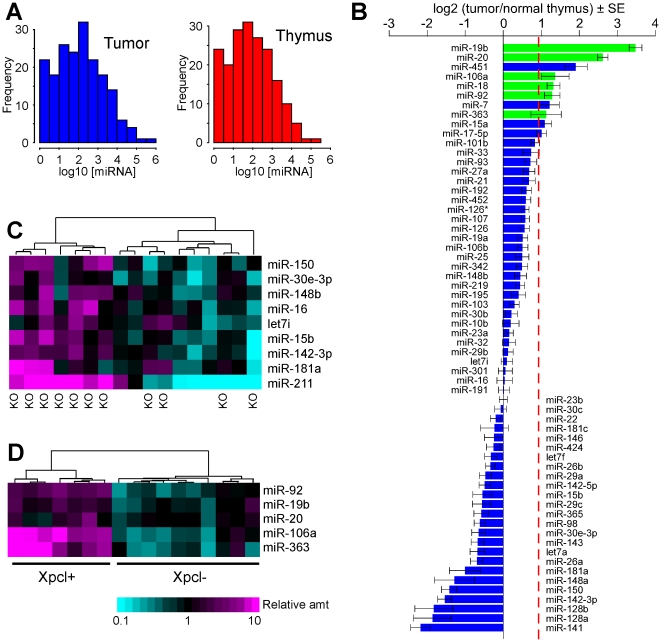
Expression of miRNA in M-MuLV lymphomas. The overall abundance and distribution of expression of individual miRNA species in (A) lymphomas, compared to normal thymus, is similar but varies over a wide range. Concentration is expressed as copies per 10 pg or input RNA. Of the 62 miRNA with expression levels>250 copies/cell (B) the majority show increased expression in tumors. (Levels displayed as the fold change of copies per tumor cell relative to copies per normal thymocyte, on a log2 scale). All 5 miRNA from the miR-106a∼363 cluster (green) are increased in tumors. The dashed red line shows magnitude of baseline shift resulting from a mean 1.9-fold increase in total RNA in tumors vs. normal thymus. Every miRNA value below this line is reduced relative to the increased total RNA content of tumor cells. Nine miRNA altered in association with *p27^Kip1^* genotype are shown in a heatmap (C) reflecting the relative abundance of each miRNA across tumor samples. Heatmap of five miRNA (D) with changes associated with *Xpcl1* viral integration status. All 5 are increased and are members of the miR-106a∼363 cluster.

Global assessments of microRNA expression have not been reported in MuLV-induced lymphomas. In this study, we used a qPCR platform (previously described) to assess expression of 188 microRNAs. [25] The source material was T-cell lymphomas that had been induced by the M-MuLV infection of mice, with or without *p27^Kip1^* knockout mutations, in F1 hybrid (C57BL/6J × 129S4) mice, as well as normal thymus controls. Retroviral integration sites were previously mapped in these samples, and integrations at the *Xpcl1* locus were identified in tumors from *p27^Kip1^* null animals, as well as in tumors arising in mice with one or more intact copies of *p27^Kip1^*. [5], [6] (The complete dataset is given in Table S1) We first compared the global miRNA expression profile of all tumors in comparison with normal murine thymus. The abundance of specific microRNAs varied over a wide range (Figure 1A). In keeping with what has been reported in human cancers, we found that the majority of microRNAs were reduced in lymphomas relative to total RNA input. [24] However, this approach does not consider the fact that lymphoma cells have a higher total RNA content than does normal thymocytes. We therefore measured the RNA and DNA content in each tumor and compared this to normal thymic tissue. On average, the lymphomas contained 1.9-fold more RNA relative to tissue mass, and relative to genomic DNA mass. Considering that M-MuLV tumors are near diploid, we conclude that there was nearly a two-fold increase in total RNA per cell in the lymphomas. [26] When adjusting for the total RNA content per cell, we find that the number of miRNAs significantly increased in lymphomas is comparable to the number that are decreased (Figure 1B). Likewise, the sum total quantity of miRNA in lymphomas was equivalent to normal tissue (Fold change = 1.08±0.05, tumor vs. normal, mean ± SE) when considered on a per cell basis.

### miRNA associated with *p27^Kip1^* null genotype and *Xpcl1* integrations

Amongst tumor samples, we compared the global microRNA expression profiles associated with *p27^Kip1^* genotype (*p27^Kip1^* null vs. wildtype), as well as the status of viral integrations at *Xpcl1* (positive or negative). Unsupervised hierarchical clustering based on abundance of individual miRNA (not shown) did not strictly segregate tumor samples either by *p27^Kip1^* genotype or by *Xpcl1* integration. This indicates that, for the majority of miRNA, neither of these factors are the major determinant of variability. Nonetheless, some of the most striking changes in miRNA expression in lymphomas included the microRNA encoded by the miR-106a-363 cluster (Figure 1B). Using a supervised analysis algorithm (PAM), we tested the hypothesis that viral integrations at *Xpcl1* are associated with expression of a distinct set of miRNA. The median false positive discovery rate (FDR) was zero at up to 5 microRNAs but rose sharply at higher numbers. This indicates that there are only 5 microRNAs (miR-363, miR-92-2, miR-20b, miR-19b, and miR-106a) influenced by viral integration at *Xpcl1* (Figure 1D). Sequence of a sixth microRNA, miR-18b, also lies in the cluster but it was not increased in *Xpcl1*+ tumors relative to *Xpc1*- tumors. Thus, the increased miR-18 levels noted in tumors (Figure 1B), may be due to expression from paralogous miR-17∼92 cluster. One potential mechanism of cooperation would be if *p27^Kip1^* deletion increased *Xpcl1* expression, e.g. by increasing expression following retroviral gene insertions or by increasing the miRNA stability. Therefore, we asked whether homozygous deletion of *p27^Kip1^* was associated with altered miRNA expression in tumor samples. A supervised analysis (Figure 1C) suggests that 9 microRNAs were associated with the *p27^Kip1^* null genotype, with considerable variability (FDR 30%). However, this list of miRNAs does not include those from the miR-106a∼363 cluster. Likewise, limiting the analysis to tumors without *Xpcl1* integrations, also does not identify miR-106a∼363 microRNA associated with the absence of *p27^Kip1^* (not shown). Thus, *p27^Kip1^* deletion does not appear to augment miR-106a∼363 expression in lymphomas, but it may induce increases in other miRNAs.

### Gene expression in M-MuLV induced tumors

**Figure 2:**
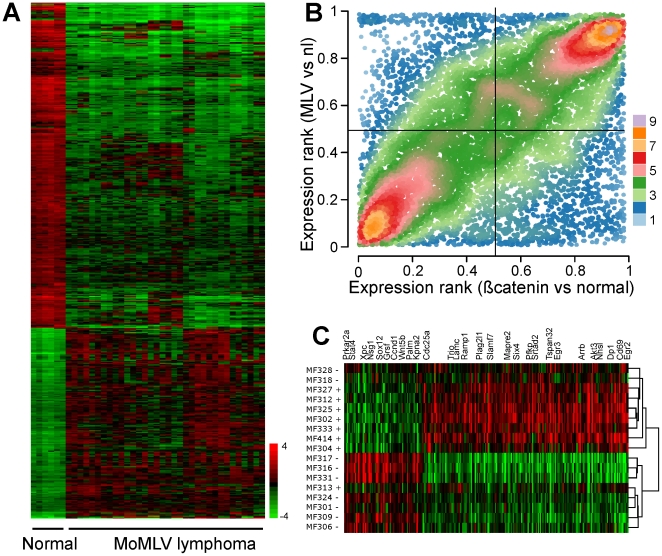
Patterns of mRNA expression in M-MuLV induced lymphomas. A heatmap (A) of the mRNA expression in 22 samples, showing both lymphomas (right) compared to normal thymus (left). The top 5000 genes (in terms of statistical significance) that display altered expression in tumors compared to normal thymus are shown. Expression levels are relative to gene-specific means, and are displayed log2 scale of microarray spot intensities (green  =  decreased, red  =  increased). A strong association of gene expression (B) in M-MuLV-induced lymphomas and ß-catenin-induced lymphomas is seen in a plot of expression rankings. The 9804 genes with the highest levels of expression are shown color coded by plot density (left lower quadrant  =  reduced gene expression in tumors on both platforms, right upper quadrant  =  increased expression). An even distribution of points across all four quadrants would be expected if there is no association in tumor gene expression between the two tumor types. A heatmap of 250 genes (C) whose expression is most strongly associated with tumors containing *Xpcl1* viral integration (+) vs. tumors without *Xpcl1* integration (−). Gene names are given for selected *Xpcl1* predicted target genes.

Because gene expression profiles have not been reported for M-MuLV induced lymphomas, we compared the general pattern of gene expression in tumors compared to normal thymic tissue. Our analysis normalized expression values within samples, as a function of both expression intensities, and 2-dimensional feature locations. [27] A supervised analysis (PAM) was used to identify genes differentially expressed in tumors (Figure 2) compared to normal thymus. In addition, tumors showed more heterogeneity of expression compared to normal tissue. In order to determine whether the pattern of expression seen in M-MuLV tumors was similar to other T-cell lymphomas we compared our data to that previously reported for T-cell lymphomas arising in mice with a ß-catenin mutation. [28] We observed a high degree of correlation across the two data sets, which is shown in Figure 2B, a plot of expression rankings. The correlation is readily apparent as an increased density of points in the right upper and left lower quadrants (R^2^ = 0.45, p<2×10^−16^ Pearson correlation). In a gene ontology assessment, the biological function terms for DNA and protein metabolism and the cell division cycle, were enriched amongst genes with high levels of expression in tumors on either platform. In contrast, T-cell function and intracellular signaling terms were enriched in the genes with reduced expression in lymphomas (Table S2). We refer to this altered pattern of gene expression as the lymphoma expression phenotype.

### Gene expression profiles of *p27^Kip1^* null and *Xpcl1*+ tumors

**Figure 3:**
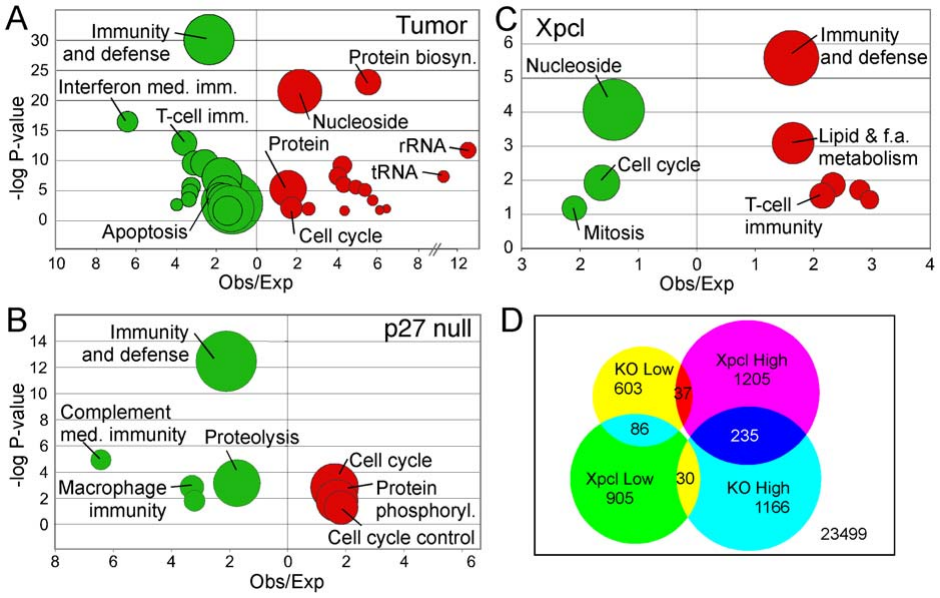
Gene expression associated with *Xpcl1* integration and *p27^Kip1^* null genotype. Biological function classes of the top 2500 genes associated with M-MuLV tumors vs. normal thymus (A) are shown in a circle plot with statistical significance plotted on the Y-axis and relative enrichment (observed/expected) plotted on the X-axis. Genes showing increased expression are shown (red) to the right and those with decreased expression (green) are plotted left of the Y-axis (P-values, binomial test). Circle diameters are proportional to the number of genes observed in each group. Within tumor samples, the functional classification of genes associated with (B) *p27^Kip1^* null genotype or (C) *Xpcl1* integrations are shown. A Venn diagram (D) shows limited overlap amongst genes exhibiting decreased expression in association with *Xpcl1* integration (*Xpcl1* Low) and simultaneously showing increased expression in *p27^Kip1^* null tumors (KO High) (n = 30). Likewise, there is little overlap between the *Xpcl1* High vs. KO Low sets (n = 37).

**Figure 4:**
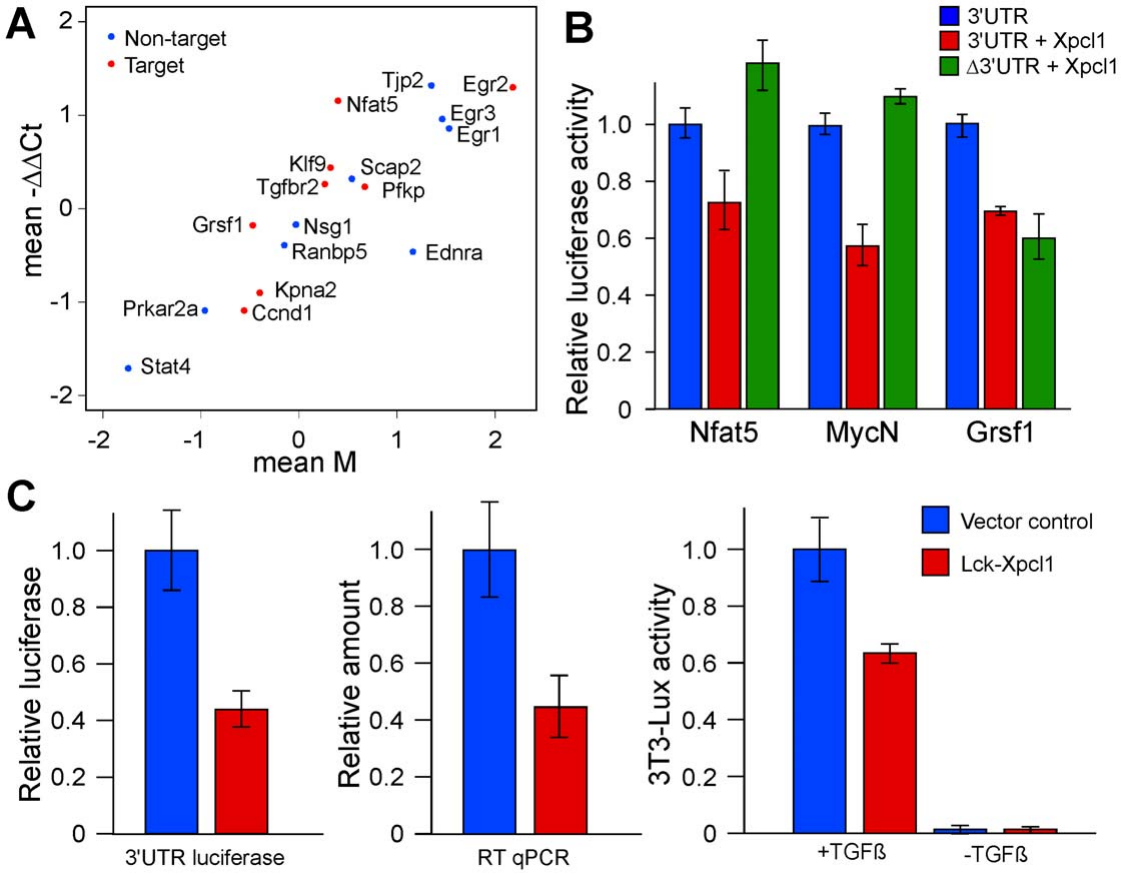
Effect of *Xpcl1* on target gene expression. (A) Differences in mRNA expression was measured by qPCR (mean -ΔΔCt) for 17 genes and plotted as the mean difference in PCR cycle number of *Xpcl1* positive vs. *Xpcl1* negative tumors. This correlated with the changes in genes expression noted on microarrays (mean M, log2(tumor/normal)). Genes with varying levels of change in association with *Xpcl1* integration are shown, some of which are predicted targets of miR-106a∼363 (red) and others which are not (blue). Comparison of luciferase activity (B) in reporter constructs containing 3'UTR sequences of miR-106a∼363 predicted target genes: *Nfat5*, *N-myc*, and *Grsf1*. Each 3'UTR reporter construct was cotransfected with either pCIG-*Xpcl1* or empty pCIG vector. *Xpcl1* was also transfected with reporter constructs with mutations in the seed region of the predicted miRNA target site (Δ3'UTR). *Xpcl1* expression reduces luciferase expression associated with the 3'UTR of *Tgfbr2* (C), and results in reduced *Tgfbr2* mRNA levels as measured by qPCR. *Xpcl1* reduces downstream signaling in response to TGF-beta (right panel C) as measured by activity of the 3T3-Lux reporter construct. (Barplots are means ± SE).

To determine whether expression of specific classes of genes is associated with *p27^Kip1^* genotype, we compared the microarray expression profiles of lymphomas from *p27^Kip1^* null animals to tumors from mice with one or two copies of *p27^Kip1^*. We ranked genes according to their PAM score, then tested for enrichment of gene ontology terms for the top 10% of genes altered in *p27^Kip1^* null tumors, using the Panther gene classification system. [29] In this analysis, statistical significance is a function of both the relative enrichment of classification terms and the size of the gene class, so we displayed results in circle plots (Figure 3A–C) showing all three parameters. Interestingly, the classes of genes enriched in *p27^Kip1^* null tumors were similar to those enriched in tumors in general (Figure 3A,B and Table S2), suggesting that the *p27^Kip1^* null tumors had a more pronounced lymphoma expression phenotype. In a separate analysis, we compared the patterns of gene expression in tumors with *Xpcl1* integrations vs. tumors without *Xpcl1* integrations, using an identical approach. There was partial overlap between *Xpcl1* and *p27^Kip1^* genotypes, with 5 out of 8 *Xpcl1*+ tumors lacking *p27^Kip1^*, and 4 out of 9 *Xpcl1*- tumors lacking *p27^Kip1^*. For this reason, a slight resemblance between the classes of genes associated with *p27^Kip1^* loss and *Xpcl1* integration might be expected. To the contrary, the functional classes associated with *p27^Kip1^* null tumors showed the opposite pattern of expression in *Xpcl1*+ tumors. For example, the cell cycle term was enriched amongst genes with increased expression in *p27^Kip1^* null tumors, but it was also enriched amongst genes decreased in *Xpcl1+* tumors (Figure 3B,C and Table S2). The opposite was true for genes in the Immunity and Defense category. These differences were largely due to changes in non-overlapping genes, as highlighted in the Venn diagram (Figure 3D), which further suggests that this observation is not simply due to the overlapping sample groups. To confirm the accuracy of the array results we verified, by qPCR, the RNA levels of 14 genes associated with *Xpcl1* integrations. There was a strong correlation (R^2^ = 0.91, p = 5×10^−6^, Pearson correlation) between mean expression levels determined by the microarrays and the qPCR assays (Figure 4A).

### *Xpcl1* target gene expression

To test the hypothesis that miRNA expression was associated with reduced expression of predicted target genes we analyzed expression of genes that are predicted targets of microRNAs overexpressed in tumors with *Xpcl1* locus integrations, and microRNAs associated with *p27^Kip1^* genotype. Within tumors, a number of miR-106a∼363 target genes displayed reduced expression in association with *Xpcl1* integration. Of 500 *Xpcl1* predicted targets decreased in tumors with *Xpcl1* viral integrations there was relative enrichment for the terms Oncogenesis, and Cell Cycle (Table 1). We cloned the 3'UTR of four predicted target genes (Figure 4B,C), which showed varying levels of reduced expression in tumors, into a luciferase reporter constructs. This included two genes (*N-myc* and *Grsf1*) that were reduced in association with *Xpcl1* viral integration, and two genes (*Tgfbr2* and *Nfat5*) that were not. In each case, cotransfection of a genomic fragment of *Xpcl1* reduced expression of the 3'UTR reporter constructs. Mutation of miRNA binding site seed regions in the 3'UTR eliminated the effect of miR-106a∼363 on the expression of *Nfat5* and *N-myc*, but did not restore *Grsf1* expression. In the case of *Tgfbr2*, miR-106a∼363 decreased the expression level of the endogenous *Tgfbr2* mRNA and decreased TGF-beta signaling as measured by the p3TP-Lux reporter construct (Figure 4D). Still, the majority of miR-106a∼363 predicted targets did not show a significant reduction at the RNA level in tumors with viral integration at *Xpcl1* (See Figure 4A and Table S3).

**Table 1:** Biological Process of *Xpcl1* targets with reduced mRNA expression.

*Xpcl1* Targets	Observed	Expected	P-value
Oncogenesis	21	9	2.2E-02
Cell cycle	35	20	5.2E-02

Panther gene classification terms for the biological function of miR-106a∼363 predicted target genes (n = 500) that show reduced expression in tumors with *Xpcl1* viral integrations, relative to the superset of genes present on the microarray. (P-values, binomial test).

## Discussion

A principal aim of this study was to determine the pattern of miRNA and protein coding gene expression associated with *p27^Kip1^* deletion or *Xpcl1* activation in M-MuLV lymphomas. Our initial hypothesis was that the two events would be associated with the activation of genes with similar functions, or in activities complementary for tumor development. Despite the extensive use of retroviral mutagenesis in mouse models, neither miRNA nor gene expression profiles have been previously reported. Thus, we felt it was imperative to observe the general patterns of gene expression in M-MuLV induced tumors compared to normal tissue, and determine whether this profile is comparable to T-cell lymphomas induced by other mechanisms. We found a global reduction in the miRNA/ total RNA ratio in tumors compared to normal thymic tissue, which is analogous to what has been described in human cancer. [24] However, it has long been recognized that proliferating cells, in general, and cancer cells, in particular, express higher levels of total RNA. [30], [31] We therefore quantified the total RNA abundance in our M-MuLV lymphoma specimens, and found that the apparent change in global miRNA levels was entirely attributable to the increased total cellular RNA in tumors. Thus, it should not be assumed that reduced miRNA levels are a general characteristic of tumors, if the data is not adjusted to cell number. Still, in terms of biological impact, a decrease in the ratio of miRNA/total RNA may have the same implications as a drop in absolute miRNA abundance. The stoichiometry of miRNA compared to the expression of their target genes is likely a key determinant of miRNA effectiveness. In contrast to the lack of change of global miRNA levels, the pattern of individual microRNA expression was highly distinctive in tumors. A large numbers of microRNA were significantly changed (both up and down) compared to normal thymus.

We found that, of the 188 miRNA queried, the only miRNAs significantly altered in association with *Xpcl1* are members of the miR-106a∼363 cluster. This reinforces observations reported for the radiation leukemia virus and SL3-3, which were also shown to be associated with increased miR-106a∼363 miRNA expression. [8], [10] Not all of the miR-106a∼363 miRNA sequences are unique. However, the fact that we see overexpression of miR-363 (which is unique to this cluster) shows that the cluster is overexpressed. Mir-18, in contrast, is also encoded by the paralogous *miR-17∼92* cluster. We observed increased expression of miR-18 in lymphomas, compared to normal thymic tissue. However, there was no further increase in miR-18 levels in tumors with *Xpcl1* integrations, so this likely represents expression from the *miR-17∼92* paralog. The broad array of miRNAs sampled here indicates the specificity of the effect of Xpc1 integration on miR-106a∼363, and argues against a general effect on miRNA expression. Deletion of *p27^Kip1^* did not independently increase the expression of miR-106a∼363, but instead may have influenced expression of a separate group of miRNAs. This argues against the hypothesis that p27Kip1 cooperates with *Xpcl1* simply by enhancing the expression of its constituent miRNAs.

In the case of protein coding genes, we observed a massive shift in the pattern of gene expression in M-MuLV induced T-cell lymphomas compared to normal thymic tissue. These changes are impressive when considered either on absolute terms (RNA per cell) or on relative terms (when adjusted to RNA abundance). On absolute terms, the overall abundance of RNA in tumors was 1.9× higher than in normal thymic tissue. Typically, expression data is normalized in ways that sets median gene expression values to a set value or, as in our case, sets the log ratios (sample vs. reference) to zero. Based on our raw (pre-normalized) data, 94% of all Entrez genes show significant increases in expression in tumors compared to normal thymic tissue (data not shown). Although this figure conveys the magnitude of shift in global RNA expression in tumor cells, it belies the shift in the pattern of gene expression exhibited by the tumors. In contrast, when expression data is subjected to conventional normalization (disregarding differences in RNA content per cell) we see that many genes exhibit relative decreases as well as increases in expression (Figure 2A). The functional classification of genes showing reduced expression in the lymphomas was frequently related to T-cell immunity (Figure 3A, Table S2). This is consistent with lymphomas developing a de-differentiated phenotype. Alternatively, it may reflect a reduced likelihood of up-regulating genes not essential to tumor development. Despite differences in array platforms, we observed a high level of similarity in gene expression in our M-MuLV lymphomas compared to that previously reported for lymphomas with a stabilizing mutation of ß-catenin. [28] In both cases, genes involved with RNA and protein metabolism were up-regulated; whereas genes involved with immunity showed relatively reduced expression. We conclude that the massive alteration in gene expression in M-MuLV induced tumors is a common feature of murine T-cell lymphomas and is not a peculiarity of M-MuLV tumor induction.

In comparison to other tumors, gene expression in *p27^Kip1^* null tumors showed further reduction in expression of genes involved with T-cell function (Figure 3). Thus, *p27^Kip1^* loss was associated with both shortened survival and a further deviation from the normal T-cell expression phenotype. [5] Interestingly, genes associated with *Xpcl1* retroviral integration showed the opposite pattern of expression, even though *Xpcl1* integration frequently occurred in *p27^Kip1^* null animals. Our finding of a contrasting pattern of expression associated with *Xpcl1* genotype compared to *p27^Kip1^* genotype does not support the hypothesis that loss of *p27^Kip1^* simply enhances *Xpcl1*'s expression phenotype. Because of sample number limitations, our assessment did not include a pair-wise comparison of all four subsets of tumor samples based on *p27^Kip1^* and *Xpcl1* genotypes. Such an approach might better elucidate the extent to which coincident mutation of *p27^Kip1^* modifies gene expression changes by *Xpcl1*. Still, our data suggests that deletion of *p27^Kip1^* counters an anti-oncogenic pattern of gene expression induced by *Xpcl1,* rather than enhancing an oncogenic effect.

According to the PicTar database 3,269 Entrez genes are predicted targets of at least one of the miR-106a∼363 miRNAs. Considering the large number of predicted targets, it is expected that miR-106a∼363 expression would induce a combination of oncogenic and anti-oncogenic effects. Others have demonstrated cellular activities by miR-106a∼363 (and its paralogous miRNA clusters) that are potentially pro-oncogenic. This includes the demonstration that anchorage independent growth is enhanced by miR-106a∼363 as well as direct targeting of *Mylip* and *Rbp1-like* gene expression. [10] Likewise, miR-106a∼363 and its paralogs have been shown to promote proliferation of cultured cells by targeting p21^Cip1/Waf1^ and p63. [32], [33] Thus it is somewhat surprising that our gene expression observations suggest that, in terms of its interaction with *Xpcl1*, the effect of *p27^Kip1^* loss is to overcome an anti-tumor activity of *Xpcl1*, as opposed to enhancing *Xpcl1*'s oncogenic effect.

In regards to the expression of miRNA target genes, we saw a high frequency of reduced gene expression (52%) amongst genes that are predicted targets of miR-106a∼363. However, we also observed a high frequency of reduced expression amongst miRNA target genes in general, regardless of the expression levels of their predicted effecter microRNAs. Thus, global reduction in miRNA target gene expression appears to be an incidental aspect of the tumor phenotype, although it might also result from secondary effects of a few overexpressed microRNAs. Two predicted targets of miR-106a∼363 miRNA are *N-myc* and *Grsf1*, both of which were decreased in *Xpcl1* positive vs. *Xpcl1* negative tumors. M-MuLV integrations were present at the *N-Myc* locus in 3 of the *Xpcl1* negative tumors that we assayed (not shown). This may partially explain the reduced levels of *N-Myc* that we observed in *Xpcl1* positive tumors. Additionally, forced expression of *Xpcl1* in cultured T-cells reduced expression of an *N-Myc* 3'UTR reporter construct. This effect by *Xpcl1* was eliminated by mutation of the predicted miRNA target site in the *N-Myc* 3'UTR, which indicates that the miRNAs directly target *N-Myc*. Reduced *N-Myc* expression would be expected to have an anti-proliferative effect, in part because *N-Myc* activates transcription of the Skp2 ubiquitin ligase, which in turn degrades *p27Kip1* protein. [34], [35] Thus, the targeting of *N-Myc* by *Xpcl1* is one example of an anti-oncogenic effect that may be overcome by *p27Kip1* deletion.

Not all of the predicted targets of miR-106a∼363 were consistently reduced in tumors with *Xpcl1* integrations. For example, *Tgfbr2*, the TGF-ß cell surface receptor, is a predicted target of both miR-19b and miR-106a, but was not significantly changed in tumors with *Xpcl1* integration. Nonetheless, luciferase reporter assays indicate that the 3'UTR of *Tgfbr2* does confer sensitivity to *Xpcl1* expression (Figure 4). Likewise forced expression of *Xpcl1* in cultured cells reduced both *Tgfbr2* mRNA levels and reduced signaling downstream of TGF-ß. To an extent, the discrepancy between tumor RNA expression and the reporter assay data may reflect the ability of the luciferase assay to detect the combined effect of miRNA on both mRNA stability and protein translation. However, endogenous gene expression is also subject to secondary effects and compensatory changes, which might easily overwhelm the comparatively subtle impact of miRNA. Finally, the existence of cell-type or gene-specific modifiers of miRNA function remains a possibility; so cultured T-cells may not accurately reflect the situation in tumors. Our list of miR-106a∼363 predicted target genes, exhibiting reduced expression in association with *Xpcl1* integration, shortens the set of genes likely to mediate the effect of miR-106a∼363 on the tumor phenotype. The confirmation of the oncogenic potential of *Xpcl1* and the elucidation of its pathogenic mechanism would further benefit from the *in vivo* manipulation of *Xpcl1* expression in the absence of other M-MuLV integrations. Given the complexity of gene expression in tumors, and the subtle yet widespread effects of miRNA expression, overexpression of *Xpcl1* in primary cells will likewise be important for the confirmation of physiologically relevant miR-106a∼363 targets.

## Materials and Methods

### Sample preparation and microRNA quantification

This work involved frozen tumor samples previously taken from *p27^Kip1^* knockout mice (p27−/−, p27+/−, and p27+/+) induced by neonatal Maloney Murine Leukemia Virus (M-MuLV) infection. These tumors were previously surveyed for the genomic integration sites of M-MuLV proviruses and are thus characterized with respect to the presence or absence of M-MuLV integrations at the *Xpcl1* locus (*Xpcl1* positive), and Myc family members. For this work tumor tissue was characterized as being p27null (−/−) vs. not null (+/− or +/+) and with respect to M-MuLV integration at the *Xpcl1* locus (positive or negative). [5] The prior animal work was conducted according to national guidelines (OLAW) and approved by the IACUC of the Fred Hutchinson Cancer Research Center. Animals had been observed daily and were euthanized if they developed signs of morbidity, palpable tumors greater than 1 cm. diameter, or age >1 year. These tumors were previously characterized for the presence of M-MuLV integrations at the *Xpcl1* locus. All animals were humanely euthanized with CO_2_ or anesthetic inhalation. Frozen whole thymus from uninfected animals (on the same 129S4 × C57BL/6J F1 hybrid strain background) was used for comparisons. Tissues were divided, weighed and then extracted for either DNA or total RNA. RNA was prepared with Trizol (Invitrogen) using 1.5 volumes of isopropanol to increase the efficiency of miRNA precipitation. RNA and DNA were quantified using Ribogreen (Invitrogen) fluorescence. Expression of individual miRNAs were determined using a quantitative primer-extension PCR assay (Table S1), as previously described. [36] Ct values were converted to copy numbers by interpolation on standard curves generated using single stranded mature miRNAs to calculate miRNA copies per 10 pg of input RNA, and are given in Table S1. The total RNA to DNA ratios of each tumor sample was determined by purifying total RNA and genomic DNA from weighed samples (<5 mg) under non-saturating conditions on nucleic acid biding columns (RNeasy and DNeasy, QIAgen). Standard curves with 2-fold dilutions (0.625–10 mg) of starting material demonstrated a high degree of linearity with respect to the mass of starting material (R^2^ = 0.985 for RNA, R^2^ = 0.984 for DNA). The *pamr* software package for R (R-project.org, BioConductor.org) was used to identify miRNA associated with *Xpcl1* integration (*Xpcl1*+ vs. *Xpcl1*−) and *p27^Kip1^* genotype (null vs. +/− or +/+). [37] The top miRNAs associated with *p27^Kip1^* genotype and *Xpcl1* integration are shown in heatmaps where quantities of individual miRNAs are displayed relative to the mean expression value of the same miRNAs across all samples. Hierarchical clustering was used to group samples post hoc using Cluster 3.0 software with Euclidean distance and complete linkage.

### Microarray analysis

Trizol prepped total RNA was additionally treated with DNAseI (RNAse free, Roche) and purified with RNeasy mini columns (Qiagen). RNA was amplified and labeled with Cy5 and Cy3 as previously described. [25] Labeled RNA pooled from 17 tumor samples and 3 normal thymuses formed a common reference and were co-hybridized vs. sample RNA in a two color format on duplicate custom murine oligonucleotide arrays (Agilent, GEO accession GPL8525) (n = 40 arrays. See Table S5 for complete sample descriptions). The genotypes of the tumor samples were balanced with roughly half of the samples (n = 9) being *p27^Kip1^* null, and half of the samples containing *Xpcl1* viral integrations (n = 8), 4 of which were both *p27^Kip1^* null and *Xpcl1*+. (See 5 for detailed sample descriptions). Log(2) ratios of fluorescence intensities (experimental sample vs. pooled reference) were normalized by intensity-dependent Loess followed by 2-dimensional location-dependent loess using the *marray* Bioconductor package without local background subtraction or scaling. [27] Missing (software flagged features) values were imputed by the K-nearest neighbor method, and genes associated with tumors vs. normal were determined with the PAM algorithm, using the *pamr* Bioconductor package. [38] We compared relative gene expression of tumors vs. normal, ranked by PAM-scores, to similarly processed raw data for ß-catenin induced tumors obtained from the GEO repository. [28] Both studies included normal thymus so we used this as a common reference. Platform specific bias was avoided by comparing ranks of expression data (expressed as log tumor/normal) and calculating the geometric means across multiple samples. This approach is equivalent to the Rank Product (RP) method, which was shown to be robust for comparing gene expression profiles. [39] Likewise, we analyzed our M-MuLV tumor data for genes associated with either *Xpcl1* integration or *p27^Kip1^* genotype (PAM-scores listed in Table S3). Data processing was streamlined with custom wrapper functions contained in the *ArrayFun* package (available at http://labs.fhcrc.org/fero/R/ArrayFun.html). This package includes functions, (*array.getGEO, array.readGEO),* that downloads data from these 40 microarrays, along with the sample description table, from the GEO FTP site directly into R. Both MIAME compliant raw and normalized data are available at the GEO data repository (accession GSE16005). [40] An R script that outlines for obtaining raw data and recapitulates the data processing steps is included as a supporting data file (Table S6). We used the “compare gene list” tool of the Panther Classification System (http://www.pantherdb.org) In order to determine the enrichment of biological function terms in genes of interest. [29] We input the top 2500 Entrez Gene IDs (ranked by PAM scores, above) and compared this to the entire set genes on the arrays. Terms over-represented in the genes of interest (p-values≤0.05, binomial test) are shown in Figure 3 and are listed in Table S2.

### MicroRNA target validation

129S4 mouse genomic *Xpcl1* DNA was isolated from a phage library. A 1.5 kB fragment containing the entire miR-106a∼363 miRNA cluster was subcloned into pCIG (courtesy of Andy McMahon) with the chicken B-actin promoter, the pSM30 vector (courtesy of Muneesh Tewari) and the p1026x vector with the Lck promoter (courtesy of Brian Iritani). [41]–[43] Genomic 3'UTR DNA segments for putative target genes (*N-Myc*, *Tgfbr2*, *Grsf1*, and *Nfat5*) were PCR amplified from mouse 129S4 genomic DNA, cloned into pGL3-control (Promega), a luciferase reporter with the SV40 promoter, and verified by sequencing. Three or four bases of miRNA seed sequence, in the UTR of predicted target genes, were mutated using the QuickChange Mutagenesis Kit (Agilent, #210513) with oligonucleotides, listed in Table S4. Wildtype and mutant 3'UTR reporter constructs were co-transfected (in triplicate) with pSM30-*Xpcl1* vs. empty vector and pRL-TK (Promega, renilla control) into 293T cells using Lipofectamine 2000 (Invitrogen). *Tgfbr2* 3'UTR luciferase assays compared pCIG-Xpcl1 vs. empty vector. Luciferase activity was assayed at 48 hr. and adjusted by renilla activity. To quantify the effect on *Tgfbr2* mRNA expression and TGF-beta signaling, murine SV40-180 T-cells were transfected by electroporation with either p1026x–Xpcl1 or empty vector. RNA was isolated at 48 h and *Tgfbr2* qPCR was done, as described above. To measure TGF-beta signaling the p3TP-Lux reporter of TGF-beta signaling (courtesy of William Grady) was additionally transfected. The cells were treated with porcine TGF-beta 1 (5 ng/mL, R&D Systems) starting at 24 hrs. post-transfection and luciferase activity was assayed at 48 h. [44] The transfection of each construct and its controls were performed in triplicate or quadruplicate.

## Supporting Information

Table S1 MicroRNA copy number per 10 pg input RNA.
(0.07 MB XLS)

Table S2 Biological Process of genes with altered expression in tumors.
(0.10 MB PDF)

Table S3 Expression array Gene IDs and PAM significance scores.
(0.96 MB XLS)

Table S4 Oligonucleotide sequences used for mutagenesis of the 3'UTR of miR- 106a∼363 target genes.
(0.07 MB PDF)

Table S5 Descriptions of RNA samples used for expression arrays.
(0.02 MB XLS)

Table S6 Instructions and R code for acquisition and analysis of expression array data.
(0.00 MB TXT)
