# Supplementary material for: Effect of Xpcl1 Activation and p27Kip1 Loss on Gene Expression in Murine Lymphoma
Source: PLoS One. 2011 Mar 11;6(3):e14758. doi: 10.1371/journal.pone.0014758 (PMC3055866; doi:10.1371/journal.pone.0014758)
Supplement: Table S2 — Biological Process of genes with altered expression in tumors. (0.10 MB PDF) [file pone.0014758.s002.pdf]

Supporting Table 2: *Biological Process of genes with altered expression in tumors.*

Category	Biological Process	Observed	Expected	P-value
Xpcl Down	Nucleoside, nucleotide metabolism	161	113	8.40E-05
	Cell cycle	55	33	1.19E-02
	Mitosis	26	12	6.51E-02
Xpcl Up	Immunity and defense	129	79	2.57E-06
	Lipid metabolism	75	45	8.07E-04
	Fatty acid metabolism	26	11	1.35E-02
	B-cell mediated immunity	18	6	1.91E-02
	T-cell mediated immunity	28	13	2.81E-02
	Interferon-mediated immunity	15	5	3.68E-02
p27 null Down	Immunity and defense	122	57	3.41E-13
	Complement-mediated immunity	14	2	1.12E-5
	Proteolysis	73	41	6.82E-4
	Macrophage-mediated immunity	19	5	1.36E-3
	B-cell immunity	15	4	1.53E-2
p27 null Up	Cell cycle	75	46.4	1.46E-3
	Protein phosphorylation	59	34.59	1.49E-2
	Cell cycle control	38	20.63	4.90E-2
Tumors Up	Immunity and defense	225	95.45	9.26E-31
	Interferon-mediated immunity	39	6.08	4.97E-17
	T-cell mediated immunity	56	15.61	1.82E-13
	Cytokine mediated signaling pathway	53	17.18	4.40E-10
	Ligand-mediated signaling	70	27.09	4.85E-10
	Cell communication	142	83.07	1.11E-07
	Macrophage-mediated immunity	31	9.53	3.51E-06
	Cytokine mediated immunity	26	7.88	3.50E-05
	Cell structure and motility	113	71.36	5.52E-05
	Apoptosis	65	35.64	1.50E-04
	JAK-STAT cascade	21	6.23	4.75E-04
	Intracellular signaling cascade	98	61.01	7.17E-04
	Signal transduction	336	276.97	2.22E-03
	MHCI-mediated immunity	14	3.53	3.87E-03
	Oncogenesis	50	28.51	4.50E-03
	Mesoderm development	66	38.79	4.90E-03
	Cell proliferation and differentiation	90	61.38	8.48E-03
	Developmental processes	189	149.48	1.75E-02
	Lipid, fatty acid and steroid metabolism	78	54.48	4.01E-02
Tumors Down	Protein biosynthesis	59	10.6	1.26E-23
	Nucleoside metabolism	174	80.46	3.90E-22
	rRNA metabolism	24	1.82	4.47E-17
	DNA metabolism	33	7.7	1.02E-09
	Pre-mRNA processing	29	7.14	6.58E-08
	tRNA metabolism	13	1.2	7.38E-08
	mRNA splicing	23	5.3	1.71E-06
	DNA repair	19	3.84	4.45E-06
	Protein metabolism and modification	120	76.01	8.44E-06
	DNA replication	16	2.96	1.30E-05
	Translational regulation	11	1.9	7.14E-04
	Cell cycle	42	24.04	1.31E-02
	Amino acid metabolism	16	6.15	1.88E-02
	Amino acid activation	7	1.08	1.92E-02
	Pyrimidine metabolism	7	1.14	2.64E-02
	DNA recombination	7	1.14	3.60E-02
	Nuclear transport	9	2.05	3.96E-02

Genes with highest PAM scores were compared to the overall set of genes present on the array using Panther Biological Processes. Ontology enrichment was assessed separately for genes showing increased expression (Xpcl1 up) from those decreased (Xpcl1 down). Likewise gene expression in tumors associated with the p27 null genotype is shown (p27 up and p27 down). Assessment of ontologies for genes increased (Tumors Up) or decreased (Tumor down) in lymphomas vs normal thymus is also shown. For each variable (Xpcl, p27 or tumor status) the top (n=2500) genes associated with the variable were assessed. P-values are from the binomial test with Bonferroni correction for multiple testing. The data in this table are shown graphically in Figure 3.
