# Supplementary material for: Effect of Xpcl1 Activation and p27Kip1 Loss on Gene Expression in Murine Lymphoma
Source: PLoS One. 2011 Mar 11;6(3):e14758. doi: 10.1371/journal.pone.0014758 (PMC3055866; doi:10.1371/journal.pone.0014758)
Supplement: Table S4 — Oligonucleotide sequences used for mutagenesis of the 3'UTR of miR- 106a∼363 target genes. (0.07 MB PDF) [file pone.0014758.s004.pdf]

Supporting Table 4: *Oligonucleotide sequences used for mutagenesis of the 3'UTR of miR-106a~363 target genes*

Symbol	miRNA	Sequence
Grsf1	19b	ATAAGGGATGAAGCAGGAAGCATCTCATTT*at*A*t*ATCTCTCTGAGACGTG
Nfat5	106a/20b	TTGTAATACTCTATTGTGC*g*C*c*T*g*AATCATTCCAAAAAAAGCCAAGAATA
Nfat5	92a	CTGACTGATTTCAATATATAGGT*t*C*c*A*g*GTTCTGTATTTCTTTAATTGTTGTGACAC
MycN	19b	GTTTAAAAAAAAAAAAAAATCAAAAT*a*T*a*C*c*A*g*GTGGCAGTGACTGTCCGTGTGGGAAACG
MycN	106a/20b	ATTTTCATAAACATGAGGTATTTC*g*A*c*G*c*G*a*TCTAAGGTGCAGCACTAAATATATAC

Shown are the identities of miRNA and their predicted target genes. The sequences of oligonucleotides used for 3'UTR mutagenesis are shown with the seed sequences (underlined) and mutated bases (in lower case italics).
